# Editorial: Long-term effects of COVID-19 pandemic on sleep and their relationships with mental health

**DOI:** 10.3389/fpsyg.2023.1281604

**Published:** 2023-09-12

**Authors:** Federico Salfi, Gianluca Ficca, Elisabet Alzueta, Nicola Cellini

**Affiliations:** ^1^Department of Biotechnological and Applied Clinical Sciences, University of L'Aquila, L'Aquila, Italy; ^2^Department of Psychology, University of Campania L. Vanvitelli, Caserta, Italy; ^3^Center for Health Sciences, SRI International, Menlo Park, CA, United States; ^4^Department of General Psychology, University of Padova, Padua, Italy

**Keywords:** sleep disturbances, mental health, psychological symptoms, pandemic, COVID-19

In the 2020–2022 biennium, humankind faced one of the worst public health crises of all time. The SARS-CoV-2 spread posed unprecedented challenges to modern societies, pervasively reshaping the daily life of everyone. The lockdown periods and the subsequent restraining measures deeply impacted the sleep and mental health of the worldwide population (Sousa et al., 2021; AlRasheed et al., 2022), with effects that persisted in the long run (Conte et al., 2021; Salfi et al., 2021). The stressful experience of SARS-CoV-2 infection and hospitalization has been associated with alarming rates of post-traumatic symptoms among COVID-19 survivors (Nagarajan et al., 2022). Moreover, social distancing drove a large-scale transition to a new working approach named *remote working*, inevitably affecting people's sleep quality and habits (Massar et al., 2021; Salfi et al., 2022). The studies collected in the present Research Topic, each distinct in its focus, converge to offer a holistic understanding of the pandemic impact on sleep, mental wellbeing, and their intricate interplay.

Massar et al. study dives into the dynamics of remote work, meticulously tracking the sleep and activity patterns of 225 Singaporean adults across three distinct pandemic phases (from August 2021 to January 2022). The investigation showed a significant shift in sleep routines among remote workers. Bedtime and wake-up time were delayed, and sleep duration increased when people worked from home. However, these changes were coupled with a significant decrease in physical activity—a concern that warrants thoughtful consideration when defining remote working policies in the post-pandemic era.

Saalwirth and Leipold delve into the complexities of chronic stress, unveiling its relationship with wellbeing indicators. The study employed a web-based survey to explore sleep quality and psychological symptoms among 480 German adults evaluated during the third COVID-19 infection wave (March–May 2021). The findings indicated that poor sleep quality was linked to heightened psychological symptoms. Notably, the study spotlighted financial worries as the dominant predictor of sleep quality, emotional wellbeing, and life satisfaction, fearing possible long-term implications of the pandemic when considering the triggered economic crisis.

The work of Hu et al. adds to a consistent body of research addressing the relationship between sleep and the psychological wellbeing of the younger population during the COVID-19 emergency. Employing a stratified cluster sampling approach, the study highlighted how lower sleep quality was associated with emotional/behavioral symptoms and social adaptation difficulties in a large sample of 6,363 Chinese college students assessed in September–December 2021.

Finally, Rapelli et al. case study addressed a timely research topic, proposing the application of Imagery Rehearsal Therapy (IRT) to treat COVID-related nightmares in a woman with a history of SARS-CoV-2 infection and consequent intensive care unit admission. The therapy led to diminished nightmare frequency and intensity, improved sleep quality, and trauma-related positive psychological changes. Overall, the study showed a promising pathway for addressing psychological distress born from severe illness and consequent hospitalization, with large-scale implications for the millions of COVID-19 survivors showing post-traumatic symptoms (Nagarajan et al., 2022).

In May 2023, the World Health Organization declares the end of the COVID-19 emergency phase. However, as evidenced by the growing literature in which this Research Topic is inserted, the sequelae of the pandemic period could accompany us for a long time. In this view, several questions remain unsolved and further studies should be performed for understanding the far-reaching impacts of the passed emergency scenario. May remote working represent an ideal approach to tackling the sleep-loss epidemic in our society (Chattu et al., 2018; Wang et al., 2023)? Considering the well-documented bidirectionality between sleep ad psychological disturbances (Alvaro et al., 2013), which is the primary antecedent of the relationship in a pandemic context? May the promotion of healthy sleep habits and the application of sleep interventions mitigate the psychological impact of the SARS-CoV-2 infection?

Although the present Research Topic is now closed, we look forward to seeing other special issues like this born in the future. Further studies are necessary to provide a long-range overview of the inextricable relationship between sleep and mental health in the post-pandemic era.

## Author contributions

FS: Writing—original draft, Writing—review and editing. GF: Writing—review and editing. EA: Writing—review and editing. NC: Writing—review and editing.
